# Evaluation of *ALK* gene rearrangement in central nervous system metastases of non-small-cell lung cancer using two-step RT-PCR technique

**DOI:** 10.1007/s12094-017-1676-4

**Published:** 2017-05-22

**Authors:** M. Nicoś, P. Krawczyk, K. Wojas-Krawczyk, A. Bożyk, B. Jarosz, M. Sawicki, T. Trojanowski, J. Milanowski

**Affiliations:** 1 Department of Pneumonology, Oncology and Allergology, Medical University of Lublin, Jaczewskiego 8, Lublin, 20-954 Poland; 2 Department of Neurosurgery and Pediatric Neurosurgery, Medical University of Lublin, Lublin, 20-954 Poland; 3 Department of Thoracic Surgery, Medical University of Lublin, Lublin, 20-954 Poland

**Keywords:** *ALK* rearrangement, RT-PCR, IHC, FISH, CNS metastases, NSCLC

## Abstract

**Purpose:**

RT-PCR technique has showed a promising value as pre-screening method for detection of mRNA containing abnormal *ALK* sequences, but its sensitivity and specificity is still discussable. Previously, we determined the incidence of *ALK* rearrangement in CNS metastases of NSCLC using IHC and FISH methods.

**Materials:**

We evaluated *ALK* gene rearrangement using two-step RT-PCR method with EML4-ALK Fusion Gene Detection Kit (Entrogen, USA). The studied group included 145 patients (45 females, 100 males) with CNS metastases of NSCLC and was heterogeneous in terms of histology and smoking status.

**Results:**

21% of CNS metastases of NSCLC (30/145) showed presence of mRNA containing abnormal *ALK* sequences. FISH and IHC tests confirmed the presence of *ALK* gene rearrangement and expression of ALK abnormal protein in seven patients with positive result of RT-PCR analysis (4.8% of all patients, 20% of RT-PCR positive patients). RT-PCR method compared to FISH analysis achieved 100% of sensitivity and only 82.7% of specificity. IHC method compared to FISH method indicated 100% of sensitivity and 97.8% of specificity. In comparison to IHC, RT-PCR showed identical sensitivity with high number of false positive results.

**Conclusion:**

Utility of RT-PCR technique in screening of ALK abnormalities and in qualification patients for molecularly targeted therapies needs further validation.

## Introduction

Anaplastic lymphoma kinase (ALK) is a transmembrane receptor showing a tyrosine kinase activity. The ALK receptor is encoded by the *ALK* gene located on chromosome 2 that includes a breakpoint frame (2;5)(p23;q35) involved in translocations. Abnormal fusions downstream the ALK signaling pathways and influence on uncontrolled cell growth or proliferation. One of the most common *ALK* fusion partner is *EML4* gene (*echinoderm microtubule*-*associated protein*-*like 4*). Mice models harboring *EML4*-*ALK* rearrangement showed uncontrolled proliferation of a lung epithelial cell with transformation to adenocarcinoma tumors [1–4].

Anaplastic lymphoma kinase (*ALK)* gene rearrangement was described in 3–7% of non-small-cell lung cancer (NSCLC) patients and it was predominantly associated with adenocarcinoma (AD) type, early age of diagnosis (median 52 years), as well as never or light smoking history (<10 pack-years) [1–4]. *ALK* aberrations are considered as a “druggable” for ALK tyrosine kinase inhibitors (TKIs). Crizotinib could be used in first or second line of treatment of advanced NSCLC patients with *ALK* gene rearrangement; however, second generation of ALK-TKIs (ceritinib, alectinib) could be used in patients with crizotinib resistance. Clinical trials with ceritinib and alectinib in first line treatment of *ALK* rearranged NSCLC patients have been completed. Moreover, the second generation of ALK-TKIs showed a high activity in NSCLC patients with distant metastases, especially in central nervous system (CNS) metastases which may extend treatment possibilities in advanced stages of NSCLC [5–7].

Evaluation of *ALK* abnormalities in NSCLC patients is necessary in their qualification to ALK-TKIs treatment. The guidelines recommend to use screening step with immunohistochemistry (IHC) to preselect patients with ALK abnormal protein. Then, results which are assessed in IHC as a positive or an equivocal should be confirmed using fluorescence in situ hybridization (FISH) that allows to visualize the presence of *ALK* gene rearrangement [8, 9]. High concordance between IHC and FISH results shows that presence of *ALK* rearrangement influence on mRNA transcript that is observed as aberrant ALK protein expression [10–12].

To date, more than 13 variants and three *ALK* fusion partners have been found, but 90% of NSCLC is associated with three major types of *EML4*-*ALK* gene (v1: E13;A20, v2: E20;A20, and v3: E6;A20) [2–4]. Indeed, large number of fusion variants is considered as a main reason of diversity in ALK expression intensity resulting false positive or negative results of IHC method. Therefore, great promises are expected with 3′ and 5′-ends mRNA-based arrays, which based on reverse-transcription PCR (RT-PCR), allow simultaneous, independent and comparative detection of various *ALK* fusion variants [6, 7, 9, 13, 14].

Based on relevant data considering effective treatment of NSCLC patients with distant metastases using ALK-TKIs, the knowledge about ALK molecular features in CNS metastases is required. Therefore, in our previous study we used the recommended procedure based on IHC and FISH techniques to assess the ALK abnormalities in 145 CNS metastases of NSCLC [15]. In the following study we decided to extend our previous analysis and performed comprehensive assessment of presence of ALK abnormalities. The aims of the study included the examination of presence of mRNA containing abnormal *ALK* gene sequences using RT-PCR with commercially available kit and the assessment of sensitivity and specificity of RT-PCR test compared to IHC and FISH examination.

## Materials and methods

### Patients

We enrolled 145 NSCLC patients with CNS metastases who were diagnosed between 2004 and 2011. Tissue samples (formalin-fixed paraffin-embedded, FFPE) from neurosurgically resected tumors were available in all patients. Only in single patients concurrent thoracic surgery was performed, therefore, the histological materials from primary tumors was not available in all patients. Moreover, the materials from corresponding primary tumors were insufficient for molecular tests (e.g., materials from fine-needle aspiration biopsies) and for this reason samples from matched primary tumors were not evaluated. Patient demographic and clinical characteristics are summarized in Table 1. All patients were radiotherapy and chemotherapy naive and underwent routine neurosurgical procedures with a palliative manner. The median overall survival (mOS) was 13.5 months (range 0.1–78.2 months; information available from 119 patients). mOS was calculated from the diagnosis of primary or metastatic lesions to the death. The study was approved by the Ethics Committee of the Medical University of Lublin, Poland (No. KE-0254/86/2013).

**Table 1:** Characteristics of the studied group

Gender	
Male, *n* (%)	100 (69)
Female, *n* (%)	45 (31)
Age	
Median age ± SD (years)	60 ± 8.8
≥60 years, *n* (%)	72 (49.7)
<60 years, *n* (%)	73 (50.3)
Histopathology	
Adenocarcinoma, *n* (%)	82 (56.6)
Squamous cell carcinoma, *n* (%) (include 1 ADSC case)	29 (20)
Large-cell carcinoma, *n* (%)	22 (15.1)
NSCLC-NOS, *n* (%)	12 (8.3)
Smoking status	
Current smokers, *n* (%)	73 (50.4)
Former smokers, *n* (%)	21 (14.5)
Non-smokers, *n* (%)	36 (24.8)
Lack of data, *n* (%)	15 (10.3)
Performance status	
0, *n* (%)	22 (15.2)
1, *n* (%)	76 (52.4)
2, *n* (%)	31 (21.4)
3, *n* (%)	16 (11)

### Methods

#### RT-PCR

mRNA was isolated from FFPE tissue samples using RNeasy FFPE Kit (Qiagen, Germany) according to manufacturer’s protocol. For molecular analysis we used RT-PCR technique with *EML4*-*ALK* Fusion Gene Detection Kit (Entrogen, USA, Research Use Only (RUO) on Cobas z 480 real-time PCR device (Roche Diagnostic, USA).

The *EML4*-*ALK* Fusion Gene Detection Kit contains reagent for two-step analysis that combine first-strand cDNA synthesis in reverse transcription and simultaneous amplification of mutant *ALK* and reference genes in a single reaction. The kit allows the detection of nine most common *EML4*-*ALK* fusion gene variants: 1: E13-A20; 2: E20-A20; 3a: E6-A20; 3b: E6-insA20; 4: E14-(-49)A20; 5a: E2-A20; 5b: E2-(+117)A20; 6: E13;(+69)A20; 7: E14-(13)A20. The kit detects 7 variants (1–3a/b,5a/b,6) in one reaction and 2 variants (4,7) in a second reaction. However, it does not distinguish between them. The analysis is based on mutant-specific primers for amplification of mutant variants of the gene, as well as dual-labeled hydrolysis probes for detection of the amplification products. Mutant variants amplification is detected with a FAM-MHQ labeled probes and the reference gene amplification is detected with a VIC/HEX equivalent-BHQ probe. The assay is designed to preferentially amplify mutant cDNA even in samples with dominance of wild-type cDNA. The assay also amplifies an internal control gene to ensure that sufficient amount of cDNA is available for amplification.

The analysis was carried out in total volume of PCR mixture (20 µl) that contained: 4 µl of enzyme mix (5×); 1.2 µl of manganese acetate; 3 µl of primer mix; 6.8 µl of RNase/DNase free water and 5 µl of tested mRNA (sample). The analysis was performed in 96-well plates in following steps: cDNA synthesis: 55 °C—10 min; 60 °C—10 min; 65 °C—10 min and 40-cycles quantification in conditions: 95 °C—10 s and 60 °C—45 s. The negative control was determined with cDNA synthesized from mRNA isolated from peripheral blood leukocytes of healthy individuals and the positive control of the analysis was the reaction with control cDNA supplied with the assay by the manufacturer.

#### FISH verification and IHC staining

All positive results obtained in RT-PCR were re-evaluated by FISH method to visualize the presence of *ALK* rearrangement using the Vysis ALK Break Apart FISH Probe Kit (Abbot Molecular, USA), paraffin-pretreatment IV and Post-Hybridization Wash Buffer Kit (Abbot Molecular, *USA*) and fluorescence microscope (Nikon Eclipse 55i, Japan). The localization and content of tumor cells in the specimens were examined with H&E staining in serially prepared slides. Way of interpretation of FISH results was in accordance to American Food and Drug Administration (FDA) guidelines, how we previously described [15, 16]. Moreover, in all samples we performed automated IHC staining on BenchMark GX (Ventana, USA) instrument. Analysis was performed using OptiView DAB Detection Kit, Positive Rabbit Monoclonal Antibody (D5F3) and Rabbit Monoclonal Negative Control (Ventana, USA) according to manufacturer’s protocol (BenchMark GX programmed for VENTANA ALK (D5F3) CDx). The staining analysis was performed according to IASLC and Ventana (Roche, USA) guidelines how we previously described [9, 15, 17].

### Statistical analysis

Statistical analysis was performed using Statistica, version 10. Associations between ALK abnormalities occurrence and clinical factors were examined using the Fisher Chi-square test. *P* values below 0.05 were considered significant. The sensitivity and specificity of diagnostic tests were calculated and positive predictive value (PPV) and negative predictive value (NPV) were determined.

## Results

Using RT-PCR technique we identified the presence of mRNA containing abnormal *EML4*-*ALK* sequences in 21% of CNS metastases of NSCLC (30/145). The abnormalities were observed slightly more frequently in female than in male patients (33%; 15/45 vs. 15%; 15/100, respectively) and in adenocarcinoma than in squamous cell carcinoma (29%; 23/80 vs. 24%; 7/29, respectively). 21% of current or former smokers (20/94) and 17% of non-smokers (6/36) showed *ALK* gene rearrangement in RT-PCR technique. The mOS of patients who showed presence of mRNA containing abnormal *EML4*-*ALK* sequence was 10.3 months and it was insignificantly longer than in patients with negative result of RT-PCR analysis (7.3 months).

FISH analysis confirmed the presence of *ALK* gene rearrangement in only sevenpatients (4.8% of all patients, 20.7% of RT-PCR positive patients) including six patients with adenocarcinoma (7.5%; 6/80) and single patient with adenosquamous carcinoma [15]. Non-diagnostic results of FISH were obtained in ten patients. All ten patients with expression of ALK abnormal protein detected in IHC method showed the presence of mRNA with abnormal sequences of *ALK* gene detected in RT-PCR test. Among these patients, we identified six patients with positive result of FISH analysis, one case with non-diagnostic results of FISH analysis and three cases with negative result in FISH examination. Summarizing of RT-PCR, IHC and FISH results was shown in Table 2.

**Table 2:** Comparison of RT-PCR positive results to IHC and FISH results obtained in the studied group

No.	Gender	Age	Histopathology	RT-PCR	IHC	FISH
1	F	57	SCC	+	−	Non-diagnostic
2	F	53	SCC	+	−	Non-diagnostic
3	F	45	SCC	+	−	−
4	F	53	AD	+	−	−
5	M	52	AD	+	−	Non-diagnostic
6	F	59	SCC	+	−	−
7	M	64	SCC	+	+	−
8	M	59	AD	+	−	Non-diagnostic
9	F	74	AD	+	+	−
10	M	63	AD	+	−	−
11	M	60	AD	+	+	−
12	M	55	AD	+	−	Non-diagnostic
13	F	54	AD	+	+	+
14	M	50	AD	+	−	Non-diagnostic
15	F	53	AD	+	+	+
16	F	57	AD	+	−	−
17	F	73	AD	+	−	Non-diagnostic
18	M	53	SCC	+	−	−
19	M	72	AD	+	−	Non-diagnostic
20	F	61	AD	+	−	−
21	M	47	AD	+	−	−
22	F	59	AD	+	+	Non-diagnostic
23	M	59	SCC	+	−	−
24	F	58	AD	+	+	+
25	M	64	AD	+	+	+
26	M	62	AD	+	−	−
27	M	58	AD-SCC	+	+	+
28	M	61	AD	+	−	−
29	M	41	AD	+	+	+
30	F	62	AD	+	+	Non-diagnostic

In spite of non-diagnostic FISH result in the sample 30 was qualified as a ALK positive how we described previously [15]

*M* male, *F* female, *AD* adenocarcinoma, *SCC* squamous cell carcinoma, *AD-SCC* adenosquamous cell carcinoma

**Fig. 1:**
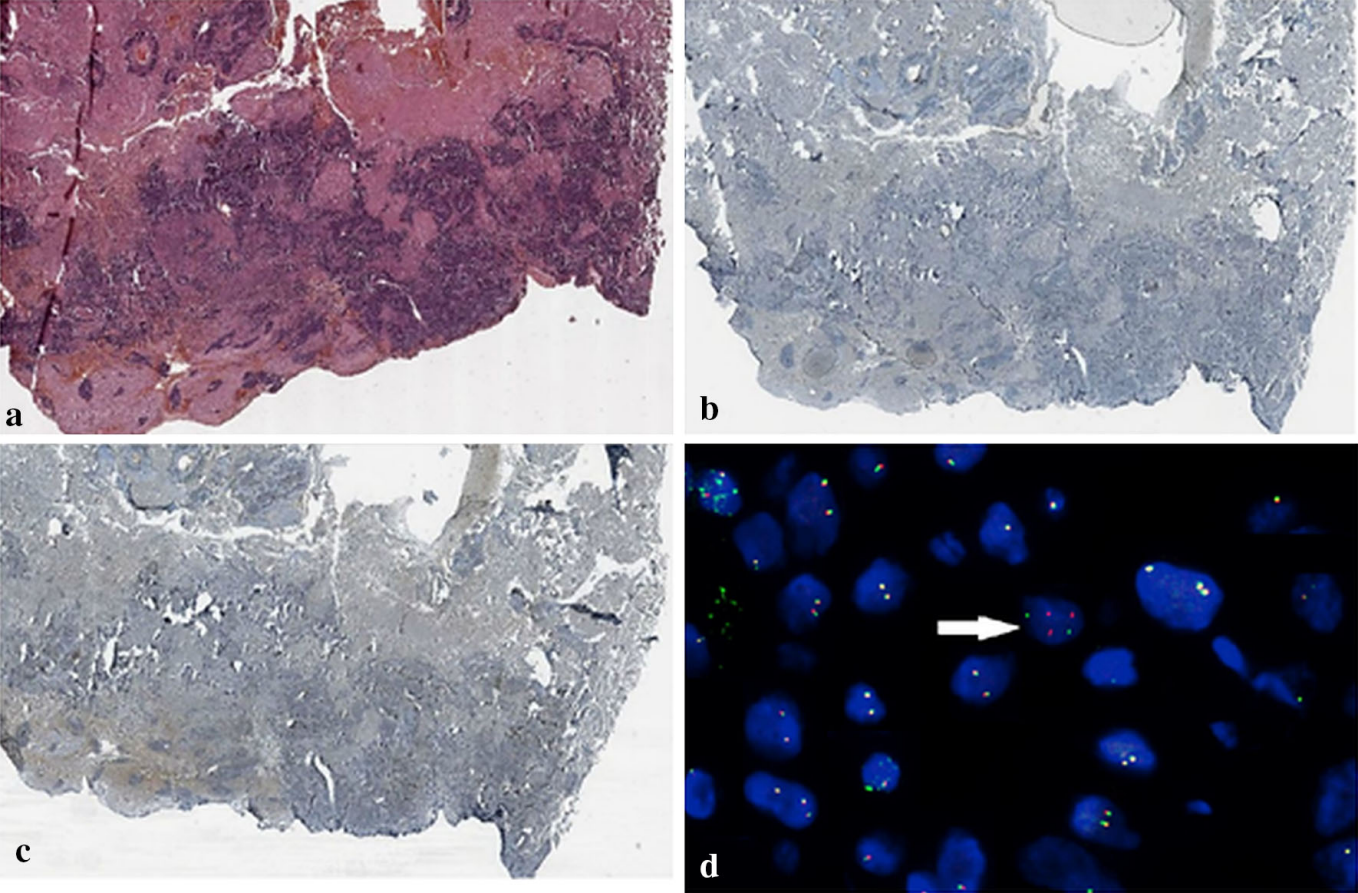
Example of lack of ALK abnormal protein expression in IHC assay and negative result of FISH assay (<15% nuclei with ALK gene rearrangement) in patients with expression of mRNA with abnormal *ALK* sequence showed in RT-PCR assay. **a** Shows H + E staining, **b** Shows negative IHC staining with Rabbit Monoclonal Negative Control, **c** shows lack of ALK abnormal protein expression with Positive Rabbit Monoclonal Antibody D5F3, **d** shows FISH result presenting single *ALK* rearranged nucleus. The *white arrow* marked single red signals

In remaining 23 samples with only RT-PCR positive results, FISH analysis gave negative results and we classified them as false positive. However, in all false positive samples, FISH analysis showed single *ALK* rearranged nucleus (less than 15% of nuclei with *ALK* gene rearrangement, Fig. 1). Therefore, RT-PCR method compared to FISH analysis achieved 100% of sensitivity and only 82.7% of specificity with PPV = 20.7% and NPV = 100%. However, IHC method compared to FISH method indicated 100% of sensitivity and 97.8% of specificity with PPV = 66.7% and NPV = 100%. However, FISH analysis generated large number of non-diagnostic results (6.9%). Therefore, we cannot be sure whether the one patient with positive results of both RT-PCR and IHC tests did not show the presence of *ALK* gene rearrangement [15].

Overall, RT-PCR technique performed with the *EML4*-*ALK* Fusion Gene Deletion Kit (Entrogen, USA) allowed us to preselect a huge cohort of patients for FISH analysis. However, in comparison to IHC, RT-PCR showed identical sensitivity with high number of false positive results.

## Discussion

In our studies, we have shown that *ALK* gene abnormalities can be detected in the CNS metastases of NSCLC and that the methods used for detection of these abnormalities have their pros and cons.

Lack of high quality of mRNA in FFPE samples, as well as too high sensitivity of RT-PCR assays are the main reasons of RT-PCR failure in screening of *ALK* gene rearrangement [13, 18]. Therefore, guidelines primarily recommend IHC and FISH techniques in selection of patients for treatment with ALK-TKIs [8]. On the other hand, comprehensive RT-PCR allows to identify the fusion partners for *ALK* gene and may have an informative value in prediction of effectiveness of treatment and patients’ survival [8, 9, 11, 13]. It was indicated that diversity of ALK fusion partners may influence on therapeutic efficacy of ALK inhibitors. In the future, RT-PCR assays could be used for monitoring of the effectiveness of treatment in mRNA isolated from liquid biopsy [3, 13, 19]. To date, utility of RT-PCR based on originally designed probes (with re-sequencing) or commercially available AmoyDx *EML4*-*ALK* Fusion Gene Detection Kit (Amoy Diagnostics, China) was described in screening for *ALK* rearrangement in NSCLC patients [11–13]. Moreover, only variants 1 and 3a/b which represent 80% of all *ALK* fusion partners were broadly described in literature and they were revealed in clinical utility [1, 4, 12].

In the following study, we used *EML4*-*ALK* Fusion Gene Detection Kit (Entrogen, USA) that allowed to find out the mRNA containing abnormal *ALK* sequences in 21% of CNS metastases from NSCLC (30/145). All positive results were observed during reaction with first set of probes covering seven fusion variants (1, 2, 3a/b, 5a/b and 6). The amplification was not observed in second set of probes covering next two variants (4 and 7) Our study was the first RT-PCR analysis of expression of mRNA containing *ALK* abnormal sequences in CNS metastases of NSCLC worldwide. However, frequency of abnormal mRNA expression remained in compliance with data obtained by Wallander et al., Zhang et al., and Teixido et al. These authors observed RT-PCR positive results in 24% (11/4), 43% (9/21) and 12.5% (25/200) of NSCLC primary tumors [7, 11, 12]. In those four studies, incidence of expression of mRNA with ALK abnormal sequences is higher than *ALK* rearrangement frequency examined by FISH method and incidence of abnormal ALK protein expression examined with IHC techniques [8, 9]. Finally, in our study all truly positive patients had diagnosed lung adenocarcinoma, and all patients with squamous cell carcinoma who had positive RT-PCR results showed IHC and FISH negative results. However, there are single data reporting the incidence of *ALK* rearrangement in lung squamous cell carcinoma (1.3%) and not-otherwise specified NSCLC (4.5%) [9].

Several studies postulated that in ALK abnormalities diagnosis, RT-PCR shows the higher sensitivity in comparison to IHC and FISH [8, 9, 11–13]. Moreover, authors indicated that sensitivity and specificity of RT-PCR compared to IHC and FISH is in the range from 95 to 100% [3, 20, 21]. We confirmed that the RT-PCR assay had 100% sensitivity, but low specificity in comparison to FISH technique. Whereas, IHC assay is characterized by higher specificity than RT-PCR when compared to FISH method. However, in our previous study we reported that tissues obtained from CNS metastases of NSCLC contained high number of macrophages, as well as nerve and glial cells, which imitate a false positive background in IHC staining. Moreover, FISH analysis gives relatively often non-diagnostic results [15].

Teixido et al. examined ALK abnormalities in 137 adenocarcinoma patients using three different methods: FISH, IHC and RT-PCR. Authors obtained positive results in 5.8, 10 and 17.5% of patients; respectively. All *ALK* rearranged patients diagnosed with FISH method were also positive in IHC and RT-PCR assays. However, six IHC-positive patients (43% of all IHC-positive patients) and 16 patients with RT-PCR positive results (67% of all RT-PCR-positive patients) had confirmed diagnosis of *ALK* rearrangement in FISH method [11]. Wallander et al. reported high concordance between RT-PCR and FISH methods (80% of consistent result), as well as between RT-PCR and IHC methods (70% of consistent result). However, FISH and IHC showed agreement in only 67% of analyzed samples [12]. On the other hand, Zhang et al. observed overall compliance results of IHC and FISH, but RT-PCR assay showed lower sensitivity than IHC method and gave large percentage of negative or equivocal results [7]. Also French multicenter study indicated 39% of false negative RT-PCR results compared to positive IHC/FISH results [22]. Shun et al. reported that the sensitivity of RT-PCR was lower than IHC assay. Furthermore, authors declared that two negative RT-PCR results showed late amplification of PCR products and they also had weak IHC expression [13]. Authors indicated different reasons for discordance between RT-PCR, FISH and IHC results. Wang et al. reported that subtle splitting of red and green FISH signals could be interpreted as false negative FISH results. They also suggested that the cross-contamination of RT-PCR samples may lead to non-specific amplification causing false positive RT-PCR results [11]. Wallander et al. suggested that discrepancies between results may be caused by various detectability of different variants of fusion genes [12].

In our study, discordant results between detectability of ALK disorders using different molecular methods might be caused by the degradation of mRNA in FFPE tissue blocks. However, more likely is the existence of a few nuclei with *ALK* gene rearrangement in examined specimens, which entitled to RT-PCR positive results and simultaneously to FISH negative results. Negative FISH results could be issued even if the existence of single nuclei with *ALK* gene rearrangement in the examined slides are visualized (Fig. 1). The slides were obtained from the patient, who was previously classified as true negative using IHC [15]. However, in following study the sample showed presence of ALK abnormal sequence in RT-PCR, therefore, we also performed FISH analysis, which presented the presence of single rearranged cells (Fig. 1d). In FISH method, when the rearrangement-positive cells rate is less than 15%, the specimen is interpreted as negative for *ALK* gene rearrangement. Indeed, RT-PCR has an extremely high sensitivity that was enough to detect single rearranged cells in analyzed material. Taking into account that only patients harboring at least 15% of rearranged cells in FISH analysis show benefit from ALK-TKIs treatment [8, 9], the utility of RT-PCR assays in qualification of NSCLC patients for targeted therapy appears to be insufficient [11].

However, cell lines studies confirmed associations between fusion variants and sensitivity to ALK inhibitors in patients with *ALK* gene rearrangement [18, 23, 24]. Yoishida et al. observed longer PFS and higher percentage of disease control after crizotinib administration in patients harboring *ALK* fusion variant 1 than in patients with other variants of *ALK* gene rearrangement [18]. Necessity of ALK fusion variants identification may increase the clinical significance of comprehensive RT-PCR analysis [12].

Summarizing, RT-PCR performed with the *EML4*-*ALK* Fusion Gene Deletion Kit (Entrogen, USA) allowed us to preselect a huge cohort of patients for diagnosis of *ALK* gene rearrangement using FISH assay. In FISH reaction, we confirmed the presence of *ALK* gene rearrangement only in samples with expression of abnormal ALK protein visualized in IHC assay and only in adenocarcinomas. RT-PCR technique showed lower specificity in comparison to IHC and FISH methods with 100% of sensitivity. Therefore, utility of RT-PCR technique both in screening patients for detection of ALK abnormalities and in qualifying patients for molecularly targeted therapies is disputable. However, our analysis allowed to detect *ALK* gene abnormalities in the CNS metastases of NSCLC using RT-PCR that was first observed in such unique material worldwide.

## Abbreviations

AD: Adenocarcinoma
ALK: Anaplastic lymphoma kinase
CNS: Central nervous system
EGFR: Epidermal growth factor receptor
FFPE: Formalin-fixed paraffin-embedded
FISH: Fluorescent in situ hybridization
IHC: Immunohistochemistry
mOS: Median overall survival
NOS: Not otherwise specified
NSCLC: Non-small-cell lung cancer
RT-PCR: Reverse transcription-polymerase chain reaction
TKIs: Tyrosine kinase inhibitors
